# Optimization of glass separating funnels to facilitate microplastic extraction from sediments

**DOI:** 10.1016/j.mex.2023.102540

**Published:** 2023-12-27

**Authors:** Mohammad Wazne, Florian Mermillod-Blondin, Manon Vallier, Stefan Krause, Nans Barthélémy, Laurent Simon

**Affiliations:** a CNRS, ENTPE, Université Claude Bernard Lyon 1, UMR 5023 LEHNA, Villeurbanne F 69622, France; b School of Geography, Earth and Environmental Sciences, University of Birmingham, Edgbaston, Birmingham B15 2TT, UK

**Keywords:** Density separation, Aquatic systems, Sediments, Polymers, Recovery rate, Stereo microscope, Glass separation funnels adapted with Mohr clamps

## Abstract

Recent studies on the distribution of microplastics in aquatic sediments have deployed different methods and devices for density separation of microplastics from sediments. However, instrument specific limitations have been noted, including their high cost, difficulty in handling, or/and the potential for elevated contamination risk due to their plastic composition. This study improves existing sediment microplastic separation techniques by modifying the commonly used conical shape glass separating funnels. The modification consists in connecting a silicone tube at the base of the funnel, whose opening and closure was manually controlled by a Mohr clamp. This adjustment made to the funnels have effectively mitigated critical clogging problems frequently encountered in density separation units. An experiment was conducted using sand-based sediment spiked with polyamide fragments to validate this method modification. Following a complete extraction protocol with the modification of separating funnels, the microplastic extraction efficiency from sediments was high with a 90% recovery rate. Based on these promising results, future studies should consider naturally diverse substrates, as recovery efficiency may be sediment-dependent.

Two key adjustments to the glass separation funnels:

• Removal of stopcocks
• Use of silicone tubes and Mohr clamps to control sediment release

## Graphical abstract

**Figure:**
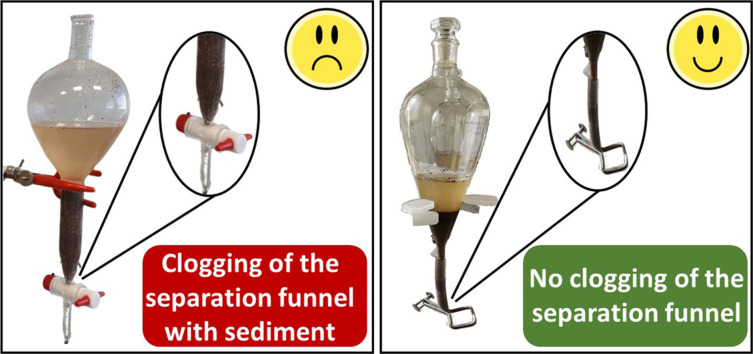

Specifications table

**Table:** 

Subject area:	Environmental Science
More specific subject area:	Microplastic contamination
Name of your method:	Glass separation funnels adapted with Mohr clamps
Name and reference of original method:	Enders K, Lenz R, Ivar do Sul JA, Tagg AS, Labrenz M. When every particle matters: A QuEChERS approach to extract microplastics from environmental samples. MethodsX. 2020 Jan 1;7:100,784. DOI: 10.1016/j.mex.2020.100784 Tophinke AH, Joshi A, Baier U, Hufenus R, Mitrano DM. Systematic development of extraction methods for quantitative microplastics analysis in soils using metal-doped plastics. Environ Pollut. 2022 Oct 1;311:119,933. DOI: 10.1016/j.envpol.2022.119933
Resource availability:	Cone-shaped glass separation funnel without stopcock Silicone tubes Mohr clamps Zinc chloride Ferrous sulfate Hydrogen peroxide (30%) Nile Red Stereo microscope

## Method details

In this work, we build on existing devices for extracting microplastics from sediments; by introducing technical adjustments to the commonly used conical shape separating funnels that will allow for easier device manipulation and yield high microplastic recovery rates. These adjustments were made in order to improve the overall efficiency of the microplastic extraction process.

## Method modification

The conical shape separating funnels used here are completely made out of glass to avoid contamination. They are low-cost and portable and have the capacity to process a significant volume of sediment all at once. However, the presence of a tap with a 0.2cm pore size opening that allows the passage of the funnel content (i.e., sediments and liquids) makes it difficult to manipulate and causes frequent clogging. This, in turn, results in disturbance of the sediments after they have settled, which makes it harder to separate microplastic particles from the sediment, which is the main aim of the entire separation step.

**Fig. 1:**
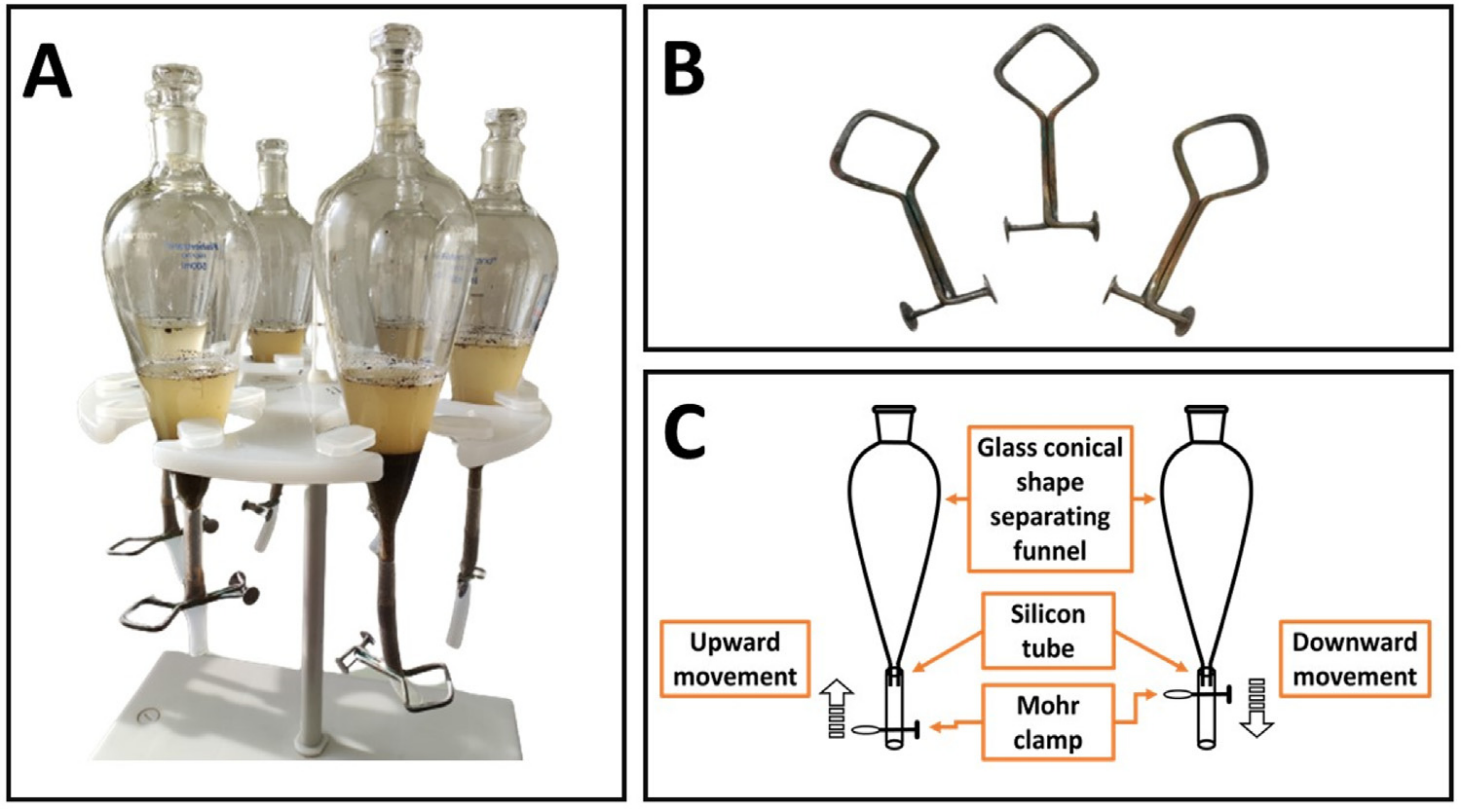
The adjusted design of cone-shaped glass separation funnels with silicone tubes and Mohr clamps (A), Mohr clamps (B), and an illustrating schema about the upward and downward move of the Mohr clamps attached adjusted to the glass funnels (C).

Because of these shortcomings of the existing approach, we designed here a 500ml cone-shaped glass separation funnel without any stop valve. The top opening, through which we sample materials are added for separation has a diameter of 2cm, while the bottom opening, through which material is removed has 0.8cm diameter (Fig. 1 A). In the new design, a tube (with a diameter of 0.9cm and a length of 12cm) made of silicone, an inorganic polymer having a higher flexibility and resistance than conventional plastics (organic polymers), is attached at the base of the funnels. Its opening and closure is manually controlled by a Mohr clamp (Fig. 1.B). The clamp also makes it possible to regulate the amount of sediment removed from the funnel during each opening of the Mohr clamp by either moving the clamp vertically upward so that sediment is ejected from the bottom portion of the silicone tube or moving the clamp down so that sediment is blocked in the tube (Fig. 1.C). This design allows to handle the sediment calmly and avoid turbulence, even with clay and other fine-grained sediments.

## Method validation

**Fig. 2:**
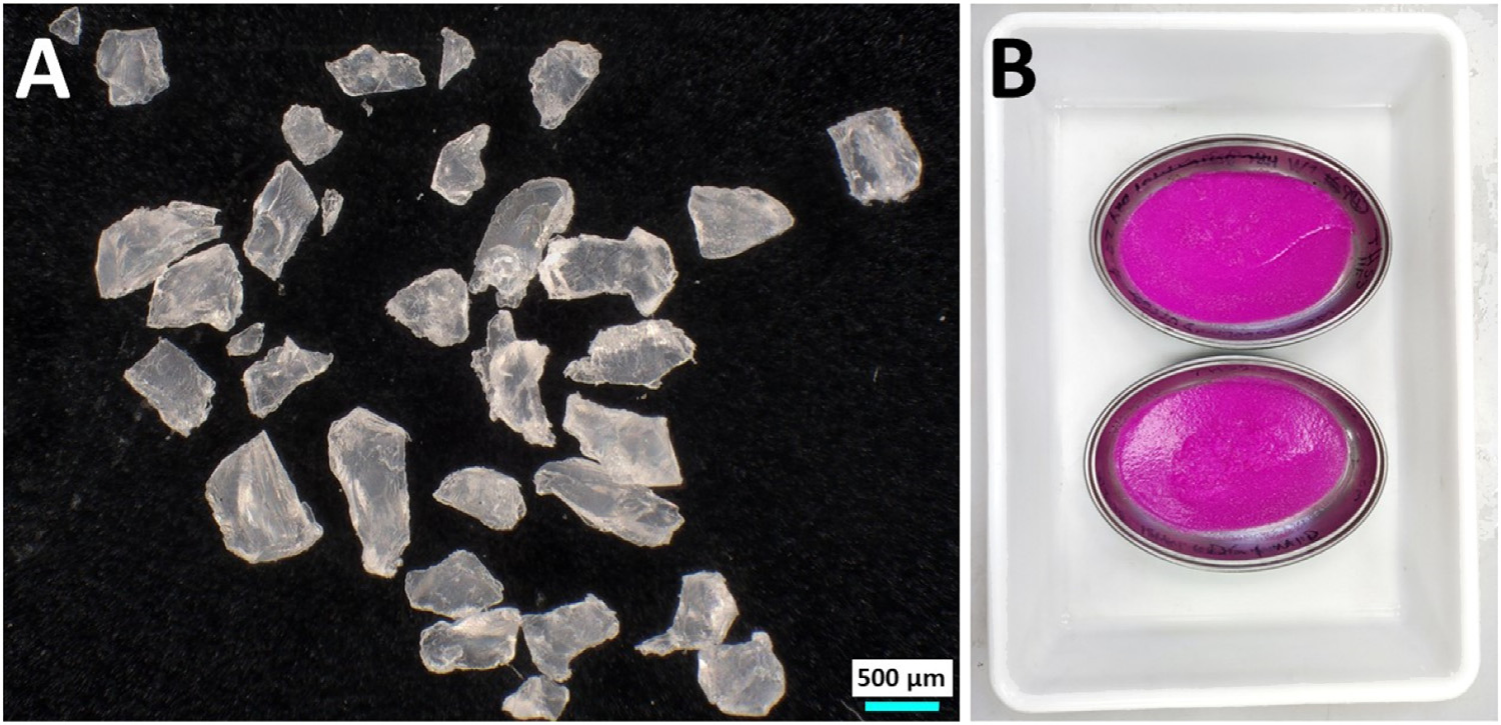
Particles of polyamide fragments observed under a digital microscope (A), and then colored with Nile Red (B).

To validate if the proposed modifications to the separation funnel design will help enhancing the handling of the manipulation with the glass separation funnels and thus obtain a sufficiently high rate of microplastic recovery, a simple but efficient test was undertaken:

First, polyamide (PA) pellets were acquired (Resinex Ltd., High Wycombe, United Kingdom), frozen at −80°C for 72 h, and then pulverized with liquid nitrogen for 20 min in a Fritsch Pulverisette 0 ball mill to form PA fragments. Then using stainless steel sieves, the resultant PA powder was sieved to retain particles smaller than 1000μm and bigger than 250µm. After confirming the size and shape of the PA particles using a digital microscope (Keyence VHX-7000), coupled with a Keyence VHX-7020 camera connected to a Keyence VH-ZST lens (Fig. 2.A), the particles were dyed with high concentrations of Nile Red (> 0.01mg ml^−1^) overnight and then oven-dried at 55°C for 48 h (Fig. 2.B), yielding fragments of PA that can be easily identified and recovered at the end of the experiment.

**Fig. 3:**
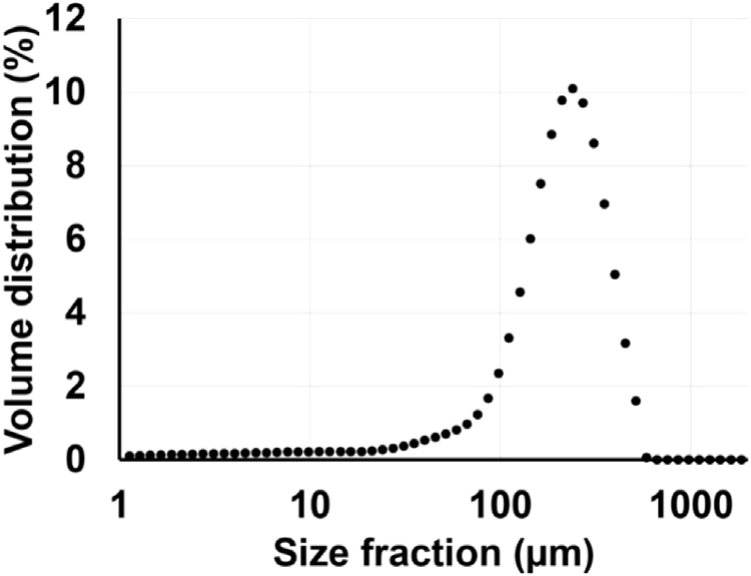
Grain size distribution of the sediment used in this experiment.

In a second step, samples of streambed sediment were taken at the Canal de Miribel on the Rhône River in the South-East of France (45°48′14.2″N 4°53′50.4″E). The particle size distribution of the sediment was determined using a laser granulometer (Mastersizer, 2000, Malvern Instrument) on three replicate samples previously treated with ultrasounds (50–60Hz, 1 min) to eliminate non-stable particle aggregates [1]. In addition, the organic matter content was quantified by the loss on ignition method, calculating the weight difference of the same sample after drying for 48 h at 55°C and burning at 550°C during 5 h. Our results indicated a dominance of fine sand particles (Fig. 3), and the presence of 0.96% of organic matter content in mass.

**Fig. 4:**
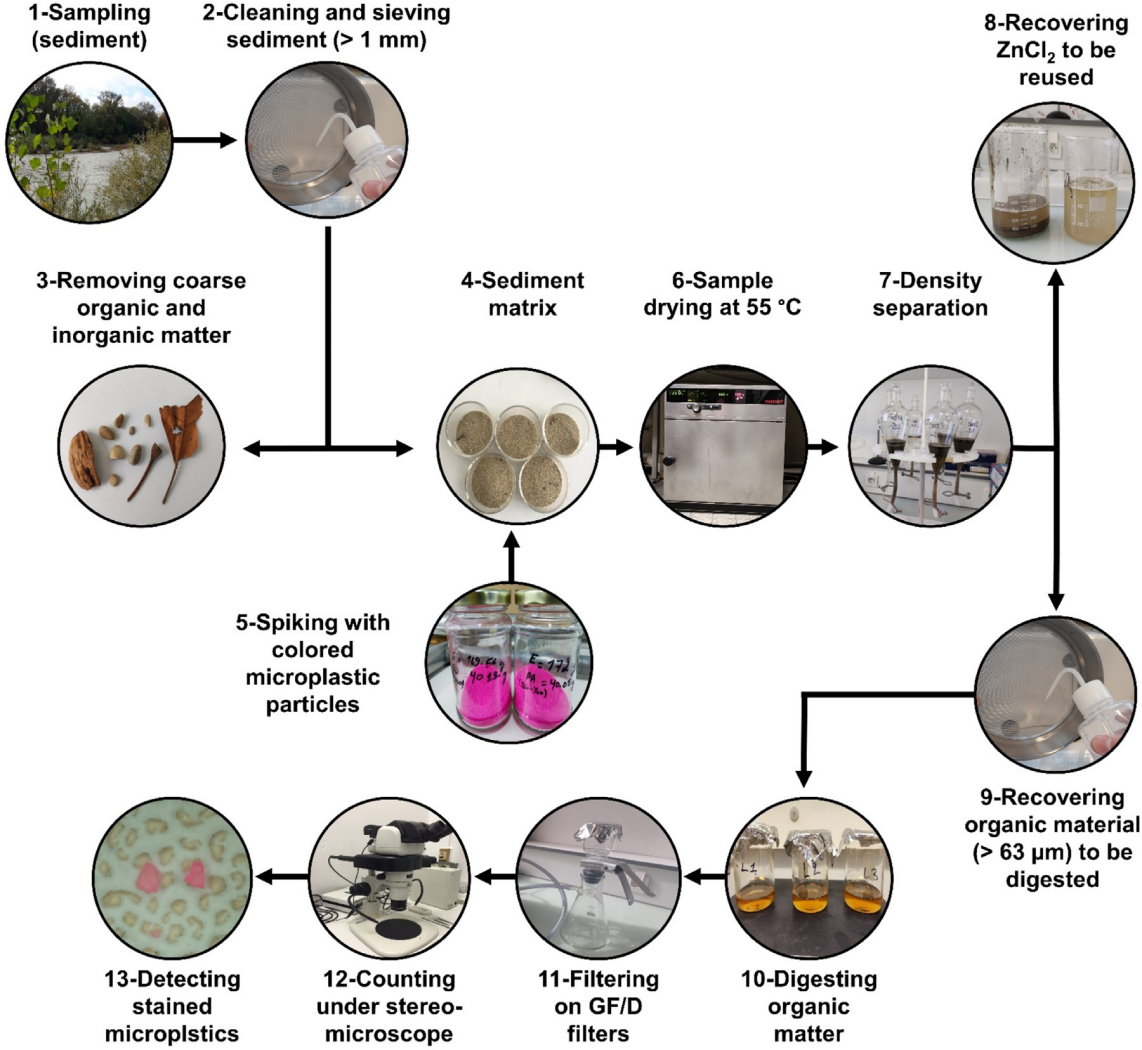
An overview of the steps of the extraction protocol of microplastics from sediments.

A sediment mixture containing a microplastic concentration of 500 particles Kg^−1^ sediment dry weight (DW) was created by spiking the sediment with colored PA fragments. Seven replicates were assembled, each containing 30g of DW sediment injected with 15 particles of the PA fragments counted under a stereo microscope (Nikon SMZ1270) to achieve the desired concentration (Fig. 4).

This spiked sediment was used to generate environmentally realistic samples so that the efficiency of the performed adjustments to the glass conical shape separating funnels could be evaluated. PA polymers were chosen as they are some of the most common plastic polymers found in the environment [2], and in particular PA fragments have been widely reported [3] in different environmental substrates. Furthermore, MP concentrations of around 500 particles Kg^−1^ DW can be considered ecologically realistic and have been found in a wide range of environmental samples [4].

The microplastic extraction protocol (Fig. 4) started by oven-drying sediment matrices for two days at 55°C. Sediment samples were then placed into glass separating funnels that had already been filled with about 100ml of a zinc chloride solution (ZnCl_2_; 1.5g cm^−3^). After that, zinc chloride solution was added until each funnel was filled to about 200ml. To ensure that plastic particles were well homogenized and mixed with the solution, the sediment matrices and the zinc chloride solution were gently agitated and shaken several times before letting the sediment settle for 24 h.

After letting the sediments settle for a full day, the Mohr clamps were gently opened and closed to release the precipitated sediments from the funnels. Without producing any turbulence, sediments were gently passed through the silicone tubes and drained off the funnels. Moving the Mohr clamp up- and downward allowed for an easy regulation of sediment discharge from the funnels. After releasing the precipitated substrate, PA particles and organic materials were left floating in the zinc chloride solution in each funnel. Following this density separation step, the remaining solutions were poured onto a 63µm mesh. The funnels were rinsed three times with ultra-pure water previously filtered through Whatman GF/D glass fiber filters (2.7µm in porosity; 47mm diameter) to ensure that all organic materials and PA particles were well collected and recovered effectively. It is necessary to point out that the use of 63µm mesh was adopted specifically for this experimental setup since the size fraction of the spiked PA particles was greater than 250µm. Nevertheless, microplastic particles smaller than 250µm may be extracted from environmental field samples using meshes with smaller pores.

Particles collected on the mesh were transferred onto a beaker using between 50 - 100ml of 30% H_2_O_2_. The beakers were then filled with 0.05M Fe(II) solution in a 10/1ml proportion. Digestion was then left to proceed for 24 h. At the following day, digested samples were poured again through a 63µm mesh and then recovered with filtered ultrapure water into new clean beakers. These samples were then filtered through Whatman GF/D glass fiber filters (2.7µm in porosity; 47mm diameter) and recovered PA particles were counted under a stereo microscope (Nikon SMZ1270 fluorescent stereo microscope) where fluorescent mode was not needed since the used PA particles were already colored and easily identified under the bright field mode.

The recovery rate (%) of the PA particles collected on the seven GF/D filters was 90.5±6.5%. The particle recovery rate was calculated by dividing the numbers of recovered particles by the number of particles added initially in each sediment replicate (Table 1). The majority of the spiked PA plastic pieces were successfully recovered in our research using the extraction process. The modifications to the glass conical shape separating funnels made it possible to operate these density separation units without clogging or disturbing the settled sediments.

**Table 1:** Number of polyamide particles recovered from the seven replicates of the spiked sediments.

Replicates	Particles recovered out of 15/30g DW sediment	Recovery rate (%)	Average	Recovery rate (%)	Standard deviation (%)
R1	13.0	86.7	13.6	90.5	6.5
R2	15.0	100.0			
R3	13.0	86.7			
R4	15.0	100.0			
R5	13.0	86.7			
R6	13.0	86.7			
R7	13.0	86.7			

Our research found a high recovery rate for PA particles, with an average extraction efficiency of 90.5% ± 6.5%. However, this recovery rate was still lower than that detected by Coppock et al. [5] (92%−98% recovery), Nakajima et al. [6] (94%−98% recovery), and Imhof et al. [7] (95.5% for microplastics smaller than 1mm). This may be due to the fact that our study assesses the recovery rate of the plastic particles after going through the steps of the entire extraction technique (Fig. 4), while earlier studies typically just tested the newly built density separation unit. Hence, it is possible that microplastic could have been lost during the digestion stage, the filtering process, or the phases of collecting PA particles on the 63µm mesh. Today, there is a lack of thorough assessments of all stages of the extraction protocol in the literature; instead, the enhanced step often stands out independently and receives more attention than the combination of all steps. However, the recovery test should not cease at the phase of modification; rather, the whole extraction protocol should be taken into account to help in the design of reliable and harmonized protocols by which researchers may refer for monitoring the actual microplastic concentrations in riverine systems.

## Additional information

Microplastic pollution of the world ocean was first reported in the 1970s, with the earliest concerns arising from North America [8,9]. Only a few years later, a number of resin pellets were found on the beaches of New Zealand, Lebanon, Spain, Canada and Bermuda [10], [11], [12], [13], [14]. By the early 21st century, Thompson provided evidence that microplastics, defined as plastic particles smaller than 5mm [15], do not simply vanish in marine ecosystems, but rather break down into smaller particles and eventually settle in sedimentary habitats [16]. Since then, microplastics have also been documented in most major surface waters, including lakes and rivers [17]. Recent research has proven that river corridors and their sediments represent long-term reservoirs of microplastics alongside marine sediments [18], [19], [20].

Microplastics in sediments pose a threat to the functioning of ecosystems [21], particularly as they can be ingested by benthic species and therefore ascend to the food chain and enter human diets [22,23]. For this reason, the development of an effective approach for detecting the distribution and concentrations of microplastics in sediments is indispensable for understanding their availability in aquatic systems and, therefore, the threat presented to aquatic animals and related ecological processes. The separation of microplastics from sediment is complicated by the presence of organic matter, which can mask microplastic particles and might mislead scientists during the identification procedures [24]. Moreover, the size, shape, and density of microplastics and sediments may make them challenging to manage. Smaller microparticles, for instance, may be more difficult to separate than larger ones, particularly in finer sediments where they might adhere to the surface of microplastics, increasing their densities and reducing the efficacy of density separation techniques [25,26]. To overcome these obstacles, a range of different methods for digesting organic materials (such as oxidation, enzymatic digestion, and acid–alkaline digestion) and separating inorganic particles (such as elutriation and density separation) have been adopted [27,28].

One of the most frequently applied methods for extracting microplastics from sediments is density separation [29]. In this method, sampled materials are separated based on their density differences using dense intermediate solutions such as sodium chloride (1.20g cm^-3^), sodium iodide (1.6–1.8g cm^-^), zinc chloride (1.5–1.8g cm^-^^3^), and zinc bromide (1.7g cm^-3^) [27,30,31]. To achieve this separation, a number of different devices and designs have been developed and deployed. Nevertheless, these devices have shown drawbacks that affect research outcomes and thus the comparability between studies [32]. Despite the fact that glass beakers are often used for this as they are readily available, the adhesion of microplastics to the glass wall might lead to a loss of plastic particles during the pouring of the supernatant. In addition, resuspension of settled sediment is highly possible [33]. This resuspension can be avoided when deploying glass funnels, yet only a little amount of sediment may be handled at a once [33]. Because of this, conical shape separating funnels are also deployed, which allow for far larger sample volumes to be treated in a single operation. However, this device is prone to frequent clogging, especially when dealing with fine sediment substrates [34,35]. The stainless-steel Munich Plastic Sediment Separator (MPSS) represents another option. It recovers a high percentage of microplastics (95.5%), but it is expensive, large in size (1.75m high), and challenging to handle [7]. Coppock et al. overcame the cost and handling issues by developing a low-cost PVC portable device [5]. These sediment microplastic isolation (SMI) units, however, contaminate the samples by introducing PVC shavings [36]. Another small portable device that can be used and is made entirely of two glass plates, is the Japan Agency for Marine-Earth Science and Technology (JAMSTEC) microplastic-sediment separator (JAMSS). Despite its high rate of microplastic recovery, this apparatus should be handled with caution as the sliding of the two glass plates can cause resuspension of the sediments from its lower to the upper tube. Microplastics have also been reported to get entangled in the silicone grease used to lubricate the plates, reducing the efficiency of recovering the plastic particles [6].-

Thus, it is essential to optimize and validate existing approaches employed for sampling, extracting and characterizing microplastics to better understand the complexity of microplastic contamination in the environment. In this technical note, our goal was to validate the adjustments made to the glass funnels by using one type of sediment matrix with only one polymer type and shape that could be easily detected under a stereomicroscope. Given that recovery rates decrease with decreasing size of microplastic particles, future studies should investigate extraction procedures with small particle fractions, particularly those smaller than 100µm [34]. In addition, other types of polymer sizes, densities, and shapes must be considered, especially since fibers are usually underestimated in the sediments because of technical limitations [26,37]. Composing a matrix that contains a heterogeneous mix of several plastic sizes, shapes, densities, polymer types, and morphologies is greatly needed.

## CRediT authorship contribution statement

**Mohammad Wazne:** Conceptualization, Methodology, Validation, Formal analysis, Investigation, Writing – original draft, Writing – review & editing, Visualization. **Florian Mermillod-Blondin:** Conceptualization, Funding acquisition, Writing – review & editing, Supervision. **Manon Vallier:** Methodology, Investigation, Visualization, Writing – review & editing. **Stefan Krause:** Conceptualization, Funding acquisition, Writing – review & editing, Supervision. **Nans Barthélémy:** Writing – review & editing. **Laurent Simon:** Conceptualization, Funding acquisition, Writing – review & editing, Supervision.

## Declaration of Competing Interest

The authors declare that they have no known competing financial interests or personal relationships that could have appeared to influence the work reported in this paper.

## Data Availability

The data will be available on request to one of the corresponding authors. Both corresponding authors have the ability to disseminate the data of this article.
